# Whole genome sequencing revealed new molecular characteristics in multidrug resistant staphylococci recovered from high frequency touched surfaces in London

**DOI:** 10.1038/s41598-019-45886-6

**Published:** 2019-08-01

**Authors:** Rory Cave, Raju Misra, Jiazhen Chen, Shiyong Wang, Hermine V. Mkrtchyan

**Affiliations:** 1 University of East London, School of Health, Sport and Bioscience, Water Lane, London, E15 4LZ UK; 2 Natural History Museum, Core Research Laboratories, Molecular Biology, Cromwell Rd, London, SW7 5BD UK; 3 Department of Infectious Disease, Huashan Hospital, Fudan University, 12 Middle Wulumuqi Rd., Shanghai, 200040 China

**Keywords:** Bacterial genetics, Mobile elements

## Abstract

The rise of antibiotic resistance (AMR) is one of the most important public health threats worldwide.Today, increasing attention is being paid to multidrug resistant staphylococci isolated from healthcare and non-healthcare environments as the treatment of these bacteria has become increasingly difficult. In this study, we compared staphylococci isolates recovered from high frequency touched surfaces from public areas in the community and hospitals in East and West London. 281 out of 600 (46.83%) staphylococci isolates recovered were multidrug resistant, of which 49 (8.17%) were *mecA* positive. There was significantly higher proportion of multidrug resistant staphylococci (P = 0.0002) in East London (56.7%) compared to West London (49.96%). The most common species identified as multidrug resistant were *S*. *epidermidis*, *S*. *haemolyticus* and *S*. *hominis*, whereas penicillin, fusidic acid and erythromycin were the most frequent antibiotics the isolates were resistant to. Whole genome sequenced of *mecA* positive isolates revealed that *S*. *sciuri* isolates carried the *mecA1* gene, which has only 84.43% homology with *mecA*. In addition, other frequently identified resistance genes included *blaZ*, *qacA/B* and *dfrC*. We have also identified a diverse range of SCC*mec* types, many of which were untypable due to carrying a novel combination of *ccr* genes or multiple *ccr* complexes.

## Introduction

Exemplified by *S*. *aureus*, staphylococci are known to cause nosocomial infections^1,2^. Due to resistance to multiple antibiotics, treatment of staphylococci infections has become increasingly difficult^3,4^. Methicillin-resistant *Staphylococcus aureus* (MRSA) and coagulase negative staphylococci (CoNS) can spread in healthcare and community-associated areas by skin to skin and skin to contaminated surfaces contacts^5–7^. Previous studies have shown those non-healthcare associated environments, including recreational beaches, public buses, residential (student) and built-up areas harbour multidrug resistant *S*. *aureus*^5,8,9^. However, studies reporting similar findings for CoNS are fragmentary^5,7,8,10–13^.

The development of antimicrobial resistance in staphylococci is due to selective pressure in the presence of antibiotics or due to stress factors in the environment^14^. Antibiotic resistance genes can be horizontally transferred to different strains and species via mobile elements such as plasmids, bacteriophages and transposons^15^. An example of this is the methicillin resistance gene *mecA* which is located on a mobile genetic element ‘staphylococcal cassette chromosome *mec* (SCC*mec*)’^16^. The *mecA* gene encodes the penicillin-binding protein 2a (PBP2a) that has a low binding affinity to all beta-lactam antibiotics^17^. The SCC*mec* is diverse in its genetic structure and to date 11 different SCC*mec* types have been characterised. SCC*mec* is determined by the combination of *mec* (A, B, C1, C2, D, E) and the chromosome cassette recombinase (*ccr*) (A1/B1, A2/B2, A3/B3, A4/B4, C1, A5/B3, A1/B6, A1/B3) complexes^18–20^. Different SCC*mec* types have evolved from two different genetic lineages, including hospital-associated and community-associated clones, however, currently, these different lineages can be found both in hospital and community environments^21^. However community-associated SCC*mec* types are generally smaller in size compared to their hospital associated counterparts^22^.

In this study, we report the differences in the proportion of multidrug resistance in CoNS staphylococci and the carriage of the *mecA* gene in isolates recovered from high-frequency hand touched surfaces of inanimate objects. Whole genome sequenced (WGS) *mecA* positive isolates revealed new molecular characteristics of these isolates.

## Results

### Sample collection

A total of 600 staphylococci isolates were recovered from general public settings and hospital public areas in East (n = 224) and West London (n = 376). 182 of 600 isolates were recovered from general public settings and 418 from public areas in hospitals. Of these 97 staphylococci were recovered from public settings from East London; 85 from public areas in West London; 127 were recovered from a hospital in East London and 291 from a hospital in West London.

### Identification of multidrug resistant staphylococci from high-frequency hand touched areas

281 multidrug resistant staphylococci isolates belonging to 11 species were identified. These included *S*. *epidermidis* (n = 75), *S*. *haemolyticus* (n = 61), *S*. *hominis* (n = 56) *S*. *saprophyticus* (n = 24), *S*. *warneri* (n = 16), *S*. *capitas* (n = 15), *S*. *cohnii* (n = 15), *S*. *sciuri* (n = 9), *S*. *aureus* (n = 5), S. *pasteuri* (n = 4) and *S*. *equorum* (n = 1). At species level *S*. *epidermidis*, *S*. *haemolyticus* and *S*. *capitis* were prevalent in West London (n = 52; n = 40; n = 13), than in East London (n = 23; n = 21; n = 2), whereas *S*. *aureus* was prevalent in East London (n = 3), than in West London (n = 2). The number of isolates of S. *hominis* (n = 31), *S*. *saprophyticus* (n = 16), *S*. *cohnii* (n = 13) and S. *pasteuri* (n = 2) recovered from these two geographic areas were largely similar. In addition, *S*. *warneri* (n = 16) was recovered from West London, but not from East London, whereas *S*. *sciuri* (n = 9) and *S*. *equorum* (n = 1) were recovered from East London, but not from West London. In total, there were 10 species of staphylococci recovered from East London, compared to 9 species from West London.

### The proportion of multidrug resistant staphylococci recovered from different public areas

281 out of 600 (46.83%) were identified as multidrug resistant staphylococci as they showed resistance to two or more antibiotics^23^. It was found that there was significantly higher proportion of multidrug resistant staphylococci (P = 0.0002) recovered from East London (56.7%) compared to those recovered from West London (49.96%) (Table 1). There was a slight significant difference (P = 0.0458) of the proportion of multidrug resistant staphylococcal isolates from public areas in the hospitals to general public settings (49.5% and 40.66% respectively) (Table 2).

**Table 1:** The proportion of multidrug resistant staphylococci and *mecA* positive isolates compared with the number of isolates analysed in East and West London and the proportion of antibiotics they were resistant to compared with the number of multidrug resistant staphylococci from East and West London.

	East London		West London		stats to test		
	Total number of samples screened (N = 224)		Total number of samples screened (N = 376)				
	N	% of total number of samples screened	N	% of total number of samples screened	% Difference	X^2^	P value
Multidrug resistant staphylococci	127	56.70	154	40.96	15.74	13.944	0.0002
*mecA* positive	24	10.71	27	7.18	3.53	2.246	0.1340
	**N**	**% MR staphylococci**	**N**	**% of MR staphylococci**	**% Difference**	**X**^**2**^	**P value**
Oxacillin	38	29.92	32	20.78	9.14	3.097	0.0784
Gentamicin R	13	10.24	13	8.44	1.79	0.268	0.6049
Gentamicin I	1	0.79	0	0.00	0.79	1.217	0.27
Muprcion R	4	3.15	8	5.19	2.05	0.706	0.4006
Muprcion I	25	19.69	4	2.60	17.09	21.879	<0.0001
Amoxicillin	33	25.98	45	29.22	3.24	0.363	0.5468
Erythromycin R	47	37.01	96	62.34	25.33	17.805	<0.0001
Erythromycin I	1	0.79	5	3.25	2.46	2.006	0.1567
Tetracycline	36	28.35	38	24.68	3.67	0.481	0.4878
Cefoxitin	29	22.83	34	22.08	0.76	0.022	0.8809
Cefepime R	7	5.51	10	6.49	0.98	0.117	0.7321
Cefepime I	2	1.57	1	0.65	0.93	0.557	0.4556
Fusidic acid	97	76.38	106	68.83	7.55	1.971	0.1603
Penicillin	102	80.31	124	80.52	0.20	0.002	0.9648
Chloramphenicol R	1	0.79	10	6.49	5.71	5.992	0.0144
Chloramphenicol I	1	0.79	2	1.30	0.51	0.17	0.6997

All chi-squared test was performed with 1 degree of freedom. R = resistance; I = intermediate resistance; MR = multidrug resistant.

**Table 2:** The proportion of multidrug resistant staphylococci and *mecA* positive isolates compared with the number of isolates analysed in general public settings and in hospitals; the proportion of antibiotics they were resistant to compared with the number of multidrug resistant staphylococci from general public settings and from hospitals.

	General public settings		Public areas in hospitals		Chi-Square test		
	Total number of samples Screened (n = 182)		Total number of samples Screened (n = 418)				
	n	% of total number of samples screened	n	% of total number of samples screened	% Difference	X^2^	P value
Multidrug resistant staphylococci	74	40.66	207	49.52	8.86	3.991	0.0458
*mecA* positive	14	7.69	33	7.89	0.20	0.007	0.9332
**Antibiotic resistance**	**N**	**% MR Staphylococci**	**N**	**% MR staphylococci**	**% Difference**	**X**^**2**^	**P value**
Oxacillin	24	32.43	46	22.22	10.21	3.097	0.0784
Gentamicin R	12	16.22	14	6.76	9.45	5.79	0.0161
Gentamicin I	0	0.00	1	0.48	0.48	0.355	0.5512
Mupirocin R	2	2.70	10	4.83	2.13	0.603	0.603
Mupirocin I	6	8.11	23	11.11	3.00	0.528	0.4674
Amoxicillin	18	24.32	60	28.99	4.66	0.591	0.4421
Erythromycin R	33	44.59	110	53.14	8.55	1.589	0.2075
Erythromycin I	1	1.35	5	2.42	1.06	0.297	0.5856
Tetracycline	27	36.49	47	22.71	13.78	5.316	0.0211
Cefoxitin	9	12.16	54	26.09	13.92	6.06	0.0138
Cefepime R	7	9.46	10	4.83	4.63	2.049	0.1523
Cefepime I	2	2.70	1	0.48	2.22	2.542	0.1109
Fusidic acid	54	72.97	149	71.98	0.99	0.027	0.8706
Penicillin	56	75.68	170	82.13	6.45	1.436	0.2308
Chloramphenicol R	1	1.35	10	4.83	3.48	1.749	0.186
Chloramphenicol I	0	0.00	3	1.45	1.45	1.081	0.2985

All chi-squared test was performed with 1 degree of freedom. R = resistance; I = intermediate resistance.

### Antibiotic susceptibility of staphylococci isolates from high-frequency hand touched surfaces

**Figure 1:**
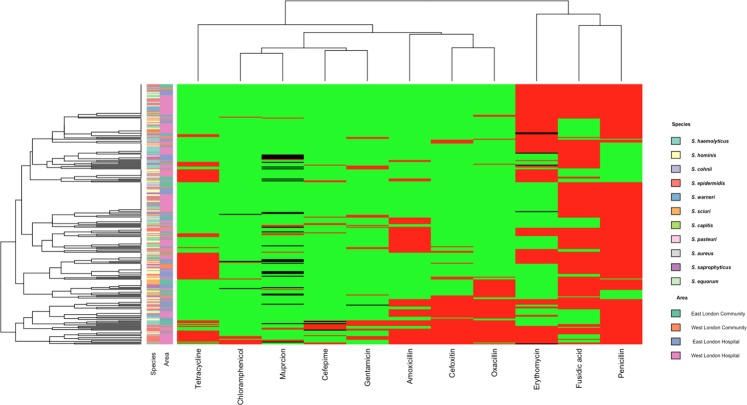
Heatmap showing hierarchical clustering of isolates antibiotic resistance profiles in comparison with the species and area they were isolated from. Red tiles represent resistance, black tiles represent intermediate resistance and green represent sensitive patterns.

All isolates were tested for their susceptibility using a panel of 11 antibiotics. Of the isolates that were shown to be multidrug resistant, 98 (34.88%) had resistance to two antibiotics; 87 (30.96%) to three antibiotics; 45 (16.01%) to four antibiotics; 15 (5.34%) to five antibiotics; 13 (4.63%) to six antibiotics; 12 (4.27%) to seven antibiotics; 9 (3.2%) to eight antibiotics and 2 (0.71%) to nine antibiotics (Fig. 1).

The most commonly found antibiotic that the staphylococci isolates were resistant to was penicillin 206 (80.42%); followed by fusidic acid 204 (72.4%) erythromycin 153 (54.45%), amoxicillin 78 (27.76%); tetracycline 74 (26.33%); oxacillin 70 (24.91%); cefoxitin 63 (22.42%); mupirocin 41 (14.59%); gentamycin 27 (9.61%); cefepime 20 (7.12%), and chloramphenicol 14 (4.98%) (Fig. 1).

Hierarchical clustering within a heat map, showed no correlation between species and area they were isolated from, to their antibiotic resistance profile (Fig. 1). The Chi-square analyses demonstrated that there was a significantly higher proportion of multidrug resistant staphylococci with erythromycin resistance (P =≤ 0.0001) and chloramphenicol resistance (P = 0.0143) from West London (62.34% and 6.49% respectively) compared to East London (37.01% and 0.79% respectively) (Table 1). The opposite was observed for mupirocin intermediate resistance with a significantly higher proportion of multidrug resistant staphylococci (P =≤ 0.0001) found in East London (19.67%) compared to West London (2.60%).

In the general public settings, there was a significantly higher proportion of isolates that had resistance to gentamycin (P = 0.00162) and tetracycline (P = 0.0211) (16.22% and 36.49% respectively) compared to public areas in hospitals (36.49% and 22.71% respectively) (Table 2). In contrast, a significantly higher proportion of multidrug resistant staphylococci (P = 0.0143) found in public areas in hospitals (26.09%) were resistant to cefoxitin compared to general public settings (12.16%).

### Detection of *mecA* gene

The *mecA* gene was identified by PCR in 49 (8.17%) of total isolates recovered. There was no significant difference in the proportion of the *mecA* gene determined in isolates recovered from East London (10.71%) compared to those recovered from West London (7.18%) (P = 0.1340), the general public settings (7.69%) and public areas in hospitals (7.18%) (P = 0.9332). Of the isolates that were *mecA* positive, 44 (62.86%) were oxacillin resistant, whereas 43 (68.25%) isolates were cefoxitin resistant. Three isolates, including one each of *S*. *hominis*, *S*. *epidermidis* and *S*. *haemolyticus* that were *mecA* positive, were shown to be sensitive to oxacillin and 6 *mecA* positive isolates, all belonging to the *S*. *sciuri* species, were sensitive to cefoxitin.

### Determination of MICs for oxacillin and cefoxitin

We have determined the MICs to oxacillin and cefoxitin for 49 isolates that carried the *mecA* gene (Table 3). Although all samples were *mecA* gene positive, only 44 CoNS isolates had MIC above the resistance breakpoints according to CSLI^24^. Five isolates, including *S*. *hominis* 372, 385, 387; *S*. *epidermidis* 465 and *S*. *haemolyticus* 361 that were *mecA* positive, were phenotypically sensitive to oxacillin. However, all five isolates were resistant to cefoxitin by zone diffusion assay. These isolates were recovered from public areas in hospitals. Neither CLSI nor BSAC recommend MIC standards for recording cefoxitin resistance^24,25^. Nevertheless, 42 out of 43 isolates in our study had MIC values of >1.5 μg/ml and were resistant to cefoxitin by a disc diffusion assay.

**Table 3:** The antibiotic resistance profile of 49 *mecA* positive isolates recovered from public areas in hospitals and general public settings.

Sample ID	species	Areas in London	Oxa	Gen	Mup	Amx	Erm	Tet	Fox	Fep	Fua	Pen	Chl	Oxa MIC (μg/ml)	Fox MIC (μg/ml)
1	*S*. *haemolyticus*	ELC	R	R	S	R	S	R	R	R	S	R	S	3	4
27	*S*. *sciuri*	ELC	R	S	S	S	S	S	S	S	R	R	S	0.5	0.75
33	*S*. *sciuri*	ELC	R	S	S	S	S	S	S	S	R	R	S	0.5	1
59	*S*. *sciuri*	ELC	R	S	S	S	S	S	S	S	R	S	S	0.75	1
74	*S*. *sciuri*	ELC	R	S	S	S	S	S	S	S	R	S	S	0.5	1
75	*S*. *sciuri*	ELC	R	R	I	S	S	S	S	S	R	S	S	0.75	1
93	*S*. *haemolyticus*	ELC	R	R	I	R	S	S	R	I	S	R	S	2	4
99	*S*. *haemolyticus*	ELC	R	R	S	R	S	R	R	S	S	R	S	3	4
105	*S*. *haemolyticus*	ELC	R	R	S	R	S	R	R	R	R	R	S	2	4
109	*S*. *sciuri*	ELC	R	S	S	S	S	S	S	S	R	R	S	1	1
207	*S*. *hominis*	WLC	R	S	S	S	R	S	R	S	R	R	S	0.5	6
208	*S*. *hominis*	WLC	R	S	S	R	R	R	R	S	R	R	S	2	6
209	*S*. *hominis*	WLC	R	S	S	R	R	S	R	S	R	R	S	1.5	6
211	*S*. *cohnii*	WLC	R	S	S	S	R	S	R	R	S	R	S	4	4
321	*S*. *epidermidis*	ELH	R	S	S	R	R	S	R	S	R	R	S	0.75	3
327	*S*. *epidermidis*	ELH	R	I	S	R	S	S	R	S	R	R	S	0.75	2
329	*S*. *epidermidis*	ELH	R	S	S	R	R	R	R	S	R	R	S	0.75	8
343	*S*. *cohnii*	ELH	R	S	S	R	R	R	R	S	R	R	S	1.5	12
349	*S*. *cohnii*	ELH	R	S	S	R	R	S	R	S	R	R	S	1.5	12
355	*S*. *epidermidis*	ELH	R	S	S	R	R	S	R	S	R	R	S	0.5	3
361	*S*. *haemolyticus*	ELH	S	S	S	R	S	S	R	S	R	R	S	0.38	4
372	*S*. *hominis*	ELH	S	S	S	S	S	S	R	S	S	R	S	0.25	6
373	*S*. *haemolyticus*	ELH	R	S	S	R	S	S	R	S	S	R	S	1	8
385	*S*. *hominis*	ELH	S	S	S	S	S	S	R	S	S	R	S	0.125	1.5
386	*S*. *hominis*	ELH	R	S	S	R	R	S	R	S	R	R	S	4	0.38
387	*S*. *hominis*	ELH	S	S	R	R	R	S	R	S	R	R	S	0.064	16
407	*S*. *epidermidis*	ELH	R	S	S	R	S	S	R	S	R	R	S	0.5	4
435	*S*. *epidermidis*	WLH	R	R	S	R	S	S	R	R	R	R	S	1	6
436	*S*. *epidermidis*	WLH	R	S	S	R	R	R	R	S	S	R	S	1.5	8
445	*S*. *haemolyticus*	WLH	R	S	I	R	R	S	R	S	S	R	R	4	4
465	*S*. *epidermidis*	WLH	S	S	S	R	R	R	R	R	R	R	R	0.38	2
475	*S*. *epidermidis*	WLH	R	S	R	S	R	S	R	R	R	R	S	2	12
479	*S*. *hominis*	WLH	R	S	S	R	R	R	R	S	R	R	S	1.5	16
492	*S*. *haemolyticus*	WLH	R	S	S	S	S	S	R	S	S	R	S	0.75	8
506	*S*. *haemolyticus*	WLH	R	S	S	R	R	R	R	R	S	R	S	4	12
538	*S*. *haemolyticus*	WLH	R	S	S	R	I	S	R	R	R	R	R	0.5	6
620	*S*. *hominis*	WLH	R	S	S	R	S	S	R	S	S	R	S	3	16
623	*S*. *hominis*	WLH	R	S	S	R	S	S	R	S	S	R	S	2	24
631	*S*. *epidermidis*	WLH	R	R	S	R	R	S	R	I	R	R	S	3	16
664	*S*. *epidermidis*	WLH	R	S	S	R	S	S	R	S	S	R	S	2	6
673	*S*. *epidermidis*	WLH	R	R	S	R	R	R	R	S	R	R	S	4	3
699	*S*. *warneri*	WLH	R	S	S	R	S	S	R	S	S	R	S	3	8
700	*S*. *warneri*	WLH	R	S	S	R	R	S	R	S	S	R	S	4	6
702	*S*. *warneri*	WLH	R	S	S	R	R	S	R	S	S	R	S	2	12
711	*S*. *epidermidis*	WLH	R	R	S	R	R	R	R	S	S	R	R	12	24
712	*S*. *epidermidis*	WLH	R	R	S	R	R	R	R	S	S	R	R	12	24
713	*S*. *epidermidis*	WLH	R	R	S	R	R	R	R	S	S	R	R	256	12
715	*S*. *epidermidis*	WLH	R	S	R	R	R	R	R	S	S	R	R	256	12
716	*S*. *epidermidis*	WLH	R	S	R	R	R	R	R	S	S	R	R	256	12

R = resistant; I = intermediate resistance, S = sensitive; Oxa = oxacillin; Gen = gentamycin; Mup = mupirocin; Amx = amoxicillin; Erm = erythromycin; tet = tetracycline; Fox = cefoxitin; Fen = cefepime, Fua = fusidic acid; Pen = penicillin; Chl = chloramphenicol ELC = East London Community; WLC = West London Community; ELH = East London Hospital; WLH = West London hospital.

### Prevalence of antibiotic genes from WGS data

The *mecA* gene was found in 43 out of 49 isolates that were whole genome sequenced. Of these none of *S*. *sciuri* isolates carried the *mecA* gene. Instead, they carried the *mecA1* gene, which had only 84.43% homology to *mecA* gene.

**Figure 2:**
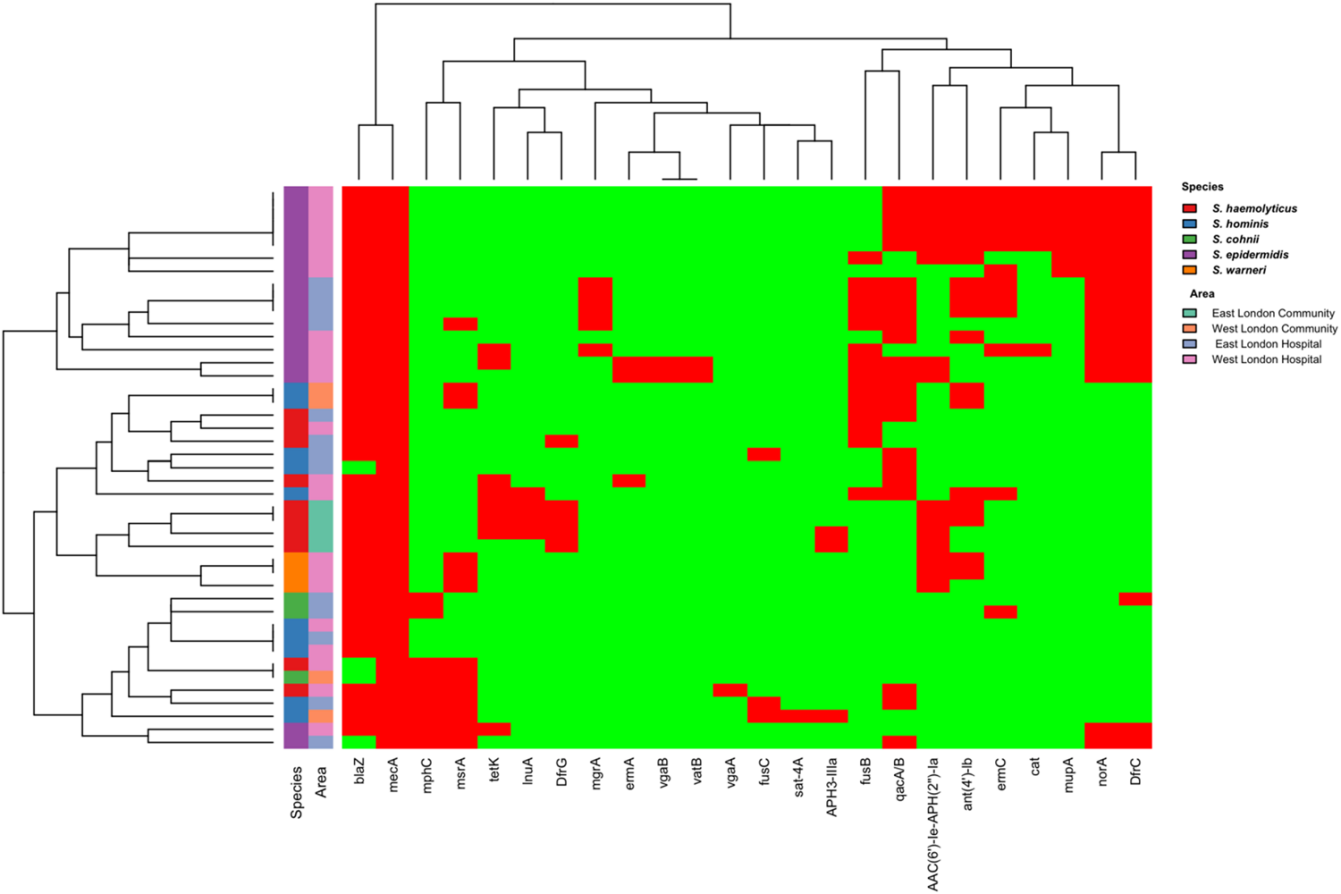
Heatmap showing hierarchical clustering of isolates resistant gene profiles in comparison with the species and area they were isolated from. Red tiles indicate presence of antibiotic resistance genes; green tiles indicate absence of resistance gene.

Apart from the *mecA*, 24 other antibiotic resistant genes were detected in 43 isolates (Fig. 2). *BlaZ* was the most commonly found resistance gene with 39 isolates (90.7%) followed by *qacA/B* with 22 (51.16%); *dfrC* with 18 (41.86%), *norA* with 17, *ant*(*4*′*)-Ib* with 17 (39.53%); *AAC(6*′*)-Ie-APH(2”)-Ia* with 15 (34.88%), *fusB* with 14 (32.56%), *msrA* with 13 (30.23%), *ermC* with 12 (27.91%), *mphC* with 9 (27.64%), *tetK* 8 (18.6%), *mupA with 7* (16.28%), *cat* with 6 (13.95%), *dfrG* with 5 (11.63%), *mgrA* with 5 (9%), *lnuA* with 4 (9.30%), *fusC* 3 (6.98%), *aph3-IIIa* 3(6.98%) and, *sat4A*, *vgaA*, *vatB* which were all found in 1 isolate (2.33%).

From these 43 isolates, 3(6.98%) had two antibiotic resistant genes, 3(6.98%) had three antibiotic resistant genes, 7 (16.28%) had four antibiotic resistant genes, 2 (4.65%) had five antibiotic resistant genes, 7 (16.28%) had six antibiotic resistant genes, 2 (4.65%) had seven antibiotic resistant genes, 3(6.98%) had eight antibiotic resistant genes, 6(13.95%) had nine antibiotic resistant genes and 5 (11.63%) had ten antibiotic resistant genes (Fig. 2).

Hierarchical clustering within a heatmap of the *mecA* isolates resistance gene profile showed a clustering of 15 of the 17 *S*. *epidermidis* isolates as well as all *S*. *warneri* isolates and *S*. *haemolyticus* from East London community (Fig. 2). Interestingly, all *S*. *epidermidis* isolates carried the *norA* and *dfrC* genes.

Barnard Exact test analysis showed that there was a significantly higher proportion of isolates with the *dfrG* gene (P = 0.0054) in East London (29.41%) compared to West London (0%) (Table 4). In addition, there was a significant higher proportion of isolates with the *cat* (P = 0.0419) and *mup* genes (P = 0.0238) in West London (23.08% and 26.92% respectfully) compared to East London (both 0%).

**Table 4:** The proportion of antibiotic resistance genes in isolates recovered from East and West London that possessed the *mecA* gene.

Antibiotic resistance genes	East London		West London		Barnard Exact test	
	Total number of samples WGS (N = 17)		Total number of isolate WGS (N = 26)			
	% of total number of samples		% of total number of samples		Difference	P value
	N	WGS	N	WGS		
*blaZ*	15	88.24	24	92.31	4.07	0.7224
*tetK*	3	17.65	11	19.23	1.58	0.9565
*ant(4*′*)-Ib*	5	29.41	12	46.15	16.74	0.3766
*AAC(6*′*)-Ie-APH(2*″*)-Ia*	4	23.53	11	42.31	18.78	0.2291
*aph3-IIIa*	2	11.76	1	3.85	7.91	0.4623
*lnuA*	3	17.65	1	3.85	13.8	0.1749
*DfrG*	5	29.41	0	0	29.41	0.0054
*DfrC*	6	35.29	12	46.15	10.86	0.7546
*fusB*	6	35.29	8	30.77	4.52	0.6665
*fusC*	2	11.76	1	3.85	7.92	0.4623
*qac*	9	52.94	13	50	2.94	0.9565
*msrA*	3	17.65	10	38.46	20.81	0.2175
*Sat4A*	0	0	1	3.85	3.85	0.4872
*mphC*	3	17.65	5	19.23	1.58	0.9565
*norA*	5	29.41	12	46.15	16.74	0.3766
*mgrA*	4	23.53	1	3.85	19.68	0.0657
*ermA*	0	0	3	11.54	11.54	0.2065
*ermC*	4	23.53	8	30.77	7.24	0.7224
*mupA*	0	0	7	26.92	26.92	0.0238
*cat*	0	0	6	23.08	23.08	0.0419
*vgaA*	0	0	1	3.85	3.85	0.4872
*vgaB*	0	0	2	7.69	7.69	0.3766
*vatB*	0	0	2	7.69	7.69	0.3766

For general public settings there was a significant higher proportion of *aph2-III* (P = 0.024), *lnuA* (P = 0.0116) and *dfrG* genes (P = 0.0031) (25%; 37.5% and 50% respectively) compared to public areas in hospitals (0%; 0% and 2.86% respectfully) (Table 5). The opposite was observed in isolates carrying the *dfrC* (P = 0.0238), and *norA* genes (P = 0.0238) with a significantly higher proportion found in public areas in hospitals (51.43% and 48.57% respectively) compared with general public settings (both 0%).

**Table 5:** The proportion of antibiotic resistant genes in isolates recovered from general public setting and public areas in hospitals that possessed the *mecA* gene.

Antibiotic resistance genes	General public settings		Public areas in hospitals		Barnard Exact test	
	Total number of samples WGS (N = 8)		Total number of isolate WGS (N = 35)			
	% of total number of samples		% of total number of samples		Difference	P value
	N	WGS	N	WGS		
*blaZ*	7	87.5	32	91.43	3.93	1
*tetK*	3	37.5	5	14.29	23.21	0.1810
*ant(4*′*)-Ib*	4	50	12	37.14	12.86	0.8026
*AAC(6*'*)-Ie-APH(2*″*)-Ia*	4	50	11	31.43	18.57	0.4519
*aph3-IIIa*	3	37.5	0	0	37.5	0.0024
*lnuA*	3	37.5	1	2.86	34.64	0.0116
*DfrG*	4	50	18	2.86	47.14	0.0031
*DfrC*	0	0	12	51.43	51.43	0.0238
*fusB*	6	25	2	25.71	0.71	1.0000
*fusC*	1	12.5	20	5.71	6.79	0.8026
*qacB*	2	25	9	57.14	32.14	0.1808
*msrA*	4	50	0	25.71	24.29	0.2078
*Sat4A*	1	12.5	7	0	12.5	0.1664
*mphC*	2	25	17	20	5	1.0000
*norA*	0	0	5	48.57	48.57	0.0238
*mgrA*	0	0	3	14.29	14.29	0.3686
*ermA*	0	0	12	8.57	8.57	0.5992
*ermC*	0	0	7	34.29	34.29	0.1664
*mupA*	0	0	6	20	20	0.1945
*cat*	0	0	1	17.14	17.14	0.2668
*vgaA*	0	0	2	2.86	2.86	0.8160
*vgaB*	0	0	2	5.71	5.71	0.8026
*vatB*	0	0	2	5.71	5.71	0.8026

### Determination of SCC*mec* types using WGS data

**Figure 3:**
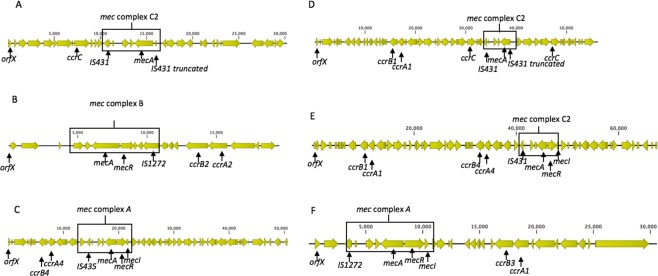
A selection of SCC*mec* structures from staphylococci isolates recovered from high-frequency touched surfaces. (**A**) Isolate 1: *S*. *haemolyticus* SCC*mec* type V; (**B**) 475: *S*. *epidermidis* SCC*mec* type IV, (**C**) 479S. *hominis* SCC*mec* type VIII; (**D**) 99*S*. *haemolyticus* with *mec* C2 complex and *ccrC*, *ccrA1/B1* complex; (**E**) 208*S*. *hominis* with a *ccrA1/B1*, *ccrB4/A4 complex* and (**F**) 211*S*. *cohnii* with a *mec* A complex and a *ccrB3/A1* complex.

The SCC*mec* types were determined in 49 *mecA* positive isolates by mapping for genetic markers from whole genome sequencing data (Table 6). 17 (34.70%) of 49 isolates harboured previously reported SCC*mec* types. These included SCC*mec* type IV (n = 11) which was exclusively found in *S*. *epidermidis* isolates from public areas in hospitals; followed by *SCCmec* type V (n = 5) found in *S*. *haemolyticus* and *S warneri* and type VIII (n = 1) found in a *S*. *hominis* isolate. The SCC*mec* element was absent in the genomes of 6 (12.45%) isolates. 5 (10.20%) isolates harboured pseudo-SCC*mec* as they had *mec* complex but lacked the *ccr* complex and 3 (6.12%) isolates had an untypable mec complex. We could not assign *SCCmec* types for the remaining 21 (42.86%) isolates as they either had a novel combination of *mec* and *ccr* complexes (n = 5); or had multiple *ccr* complexes (n = 13) or novel *ccr* complexes (n = 2); or had an untypable mec complex (n = 1). A select few of these SCC*mec* structures is illustrated in Fig. 3.

**Table 6:** The diversity of SCC*mec* types of the 49 coagulase negative staphylococci isolates recovered from public areas from the community and general public areas in hospitals.

Sample no	Area	Species	*mec* complex	*ccr* complex	SCC*mec* type
1	ELC	*S*. *haemolyticus*	C2	C	V
27	ELC	*S*. *sciuri*			No SCC*mec* element
33	ELC	*S*. *sciuri*			No SCC*mec* element
59	ELC	*S*. *sciuri*			No SCC*mec* element
74	ELC	*S*. *sciuri*			No SCC*mec* element
75	ELC	*S*. *sciuri*			No SCC*mec* element
93	ELC	*S*. *haemolyticus*	Untypable		Pseudo
99	ELC	*S*. *haemolyticus*	C2	C A1/B1	Untypable
105	ELC	*S*. *haemolyticus*	C2	C	V
109	ELC	*S*. *sciuri*			No SCC*mec* element
207	WLC	*S*. *hominis*	A		Pseudo
208	WLC	*S*. *hominis*	A	A1/B1, A4/B4	Untypable
209	WLC	*S*. *hominis*	A	A1/B1, A4/B4	Untypable
211	WLC	*S*. *cohnii*	A	A1/B3	Untypable
321	ELH	*S*. *epidermidis*	B	A2/B2	IV
327	ELH	*S*. *epidermidis*	B	A2/B2	IV
329	ELH	*S*. *epidermidis*	B	A2/B2	IV
343	ELH	*S*. *cohnii*	A	A1, A3/B3	Untypable
349	ELH	*S*. *cohnii*	A	A1, A3/B3	Untypable
355	ELH	*S*. *epidermidis*	A	C, A2/B2	Untypable
361	ELH	*S*. *haemolyticus*	C2		Pseudo
372	ELH	*S*. *hominis*	A	A1/B1	Untypable
373	ELH	*S*. *haemolyticus*	Untypable		Pseudo
385	ELH	*S*. *hominis*	A	C, A1/B3	Untypable
386	ELH	*S*. *hominis*	A	A1/B1	Untypable
387	ELH	*S*. *hominis*	C2	A1/B1	Untypable
407	ELH	*S*. *epidermidis*	C2	C, A2/B2	Untypable
435	WLH	*S*. *epidermidis*	A	C, A2/B2	Untypable
436	WLH	*S*. *epidermidis*	B	C, A3/B3/, A4/B4	Untypable
445	WLH	*S*. *haemolyticus*	A	A2/B2	IV
465	WLH	*S*. *epidermidis*	B	A1/B1	Untypable
475	WLH	*S*. *epidermidis*	A	A2/B2	IV
479	WLH	*S*. *hominis*	A	A4/B4	VIII
492	WLH	*S*. *haemolyticus*	Untypable	C B4/A4	Untypable
506	WLH	*S*. *haemolyticus*	C2		Pseudo
538	WLH	*S*. *haemolyticus*	C2	A4/B4	Untypable
620	WLH	*S*. *hominis*	A	A1/B2	Untypable
623	WLH	*S*. *hominis*	B	A1/B2	Untypable
631	WLH	*S*. *epidermidis*	B	A2/B2	IV
664	WLH	*S*. *epidermidis*	B	C A2/B2	Untypable
673	WLH	*S*. *epidermidis*	C2	C A2/B2	Untypable
699	WLH	*S*. *warneri*	C2	C	V
700	WLH	*S warneri*	C2	C	V
702	WLH	*S warneri*	B	C	V
711	WLH	*S*. *epidermidis*	B	A2/B2	IV
712	WLH	*S*. *epidermidis*	B	A2/B2	IV
713	WLH	*S*. *epidermidis*	B	A2/B2	IV
715	WLH	*S*. *epidermidis*	B	A2/B2	IV
716	WLH	*S*. *epidermidis*	B	A2/B2	IV

ELC = East London Community; WLC = West London Community, ELH = East London Hospital; WLH = West London Hospital.

### MLST typing for *Staphylococcus epidermidis*

MLST was determined for *S*. *epidermidis* isolates inferred from whole genome sequencing. 10 different sequence types (ST) were assigned to 17 *S*. *epidermidis* isolates (Table 7). ST2 was the most common (n = 5) sequence type, followed by ST66 (n = 3) and ST87 (n = 2). Two new sequence types were identified which have been assigned ST771 and ST779.

**Table 7:** MLST types of *S*. *epidermidis* isolates.

Sample No	Area	Sequence type (ST)
321	ELH	66
327	ELH	66
329	ELH	66
355	ELH	558
407	ELH	59
435	WLH	188
436	WLH	771
465	WLH	54
475	WLH	5
631	WLH	87
664	WLH	779
673	WLH	87
711	WLH	2
712	WLH	2
713	WLH	2
715	WLH	2
716	WLH	2

ELH = East London Hospital; WLH = West London Hospital.

## Discussion

Antibiotic resistance is a global public health concern. Increasingly, antibiotic resistant bacteria are emerging from different ecological niches^5,7,8,10–12^. It has been documented that surfaces in hospitals and non-hospital areas can be potential reservoirs for antibiotic resistant staphylococci, however studies comparing general public areas and that of public areas in hospitals are fragmentary^8,26^. In this study we compare the levels of antibiotic resistant staphylococci in general public areas and that of public areas in hospitals in two different geographical areas in London and provide insights into the molecular characterisation of these isolates.

281 multidrug resistant staphylococci isolates belonging to 11 species were identified in this study. The most prevalent species were *S*. *epidermidis* (n = 74) and *S*. *haemolyticus* (n = 61). *S*. *epidermidis* and *S*. *haemolyticus* have previously been reported as the most common CoNS isolated from surfaces in public settings and hospitals^26^. Interestingly, it has been demonstrated that *S*. *aureus (*n = 5) was less prevalent on high-frequency hand touched surfaces. This may be due to the fact that *S*. *aureus* is more commonly carried in the nasal passages than on hands^27^. *S*. *aureus* is the most virulent species of staphylococci and the most common cause of infection in hospitalised patients^28^. However, *S*. *epidermidis*, *S*. *hominis* and *S*. *haemolyticus* are amongst the most frequent nosocomial pathogens responsible for minor skin infections to life-threatening diseases^1,29^. In addition, community associated CoNS have also been reported to cause infections^30^.

Amongst the staphylococci isolates we detected increased susceptibilities toward penicillin (80.42%), fusidic acid (72.4%), and erythromycin (54.45%). Xu *et al*. reported increased susceptibilities toward penicillin, fusidic acid, erythromycin, and cefepime among staphylococci isolates recovered from surfaces of inanimate objects in London hotel rooms^7^. It has been reported that in primary care in England 48.8% of antibiotics prescribed were penicillin’s and 13.4% were macrolides, lincosamide and streptogramins^31^. The high usage of these antibiotics might relate to why it is common to see penicillin and erythromycin resistance from staphylococci isolates from general public settings.

Areas in East and West London harboured high levels of antibiotic resistant staphylococci in proportion to the number of isolates that were examined. Significantly higher proportion (P = 0.0002) of multidrug resistant staphylococci was observed from East London (56.7%) compared to West London (49.96%). This may be due to East London having a higher population density (9.7 thousand per square km; 2017 estimate) compared to West London (8.9 thousand per square km; 2017 estimate)^32^. Previous studies have shown that there is a linkage in population density to the development of antibiotic resistant^33^.

There was no difference in distribution of these multidrug resistant isolates at species level in two geographical areas at species level, apart from that *S*. *warneri* isolates were exclusively recovered from West London, but not from East London, whereas *S*. *sciuri* and *S*. *equorum* were recovered from East London only.

In this study, we isolated high levels of multidrug resistant staphylococci in public areas in hospitals and general public settings. Statistically, there was a significantly higher proportion (P = 0.0458) of multidrug resistant staphylococci in public areas in hospitals (49.5%) compared to that in general public settings (40.66%) which was expected due to the increase use of antibiotics in hospitals than in the community^34^. However, the proportions of multidrug resistant bacteria isolated from general public settings in our study (46.83%) were less than that reported in similar studies from a university campus in Thailand (61%) and hotel rooms in London (86%)^7,11^. In this study isolates were recovered from areas in hospitals that were accessible to the general public and not just to the hospital staff or patients. These areas included reception areas, public washrooms, corridors and lifts. The high levels of multidrug resistant staphylococci recovered from these areas in hospitals suggest a cross-contamination between community-associated and hospital-associated staphylococci.

We did not detect a significant difference in the carriage of *mecA* gene in isolates recovered from East (10.71%) and West London (7.18%) and from general public settings (7.69%) and public areas in a hospital (7.18%). The prevalence of the *mecA* gene in general public settings was less than that reported from the university campus in Thailand (20.5%) and hotel rooms in London (29.6%)^7,11^. In this study, the prevalence of the *mecA* gene in hospitals (7.89%) was also less than it was reported from a hospital in Thailand (70.1%). For the latter it was expected because of the high levels of antibiotic exposure as the isolates were recovered from the hospital wards in Thailand^26^.

Interestingly, we found that the 6 *S*. *sciuri* isolates that were resistant to oxacillin and were *mecA* positive (determined by PCR), carried a homolog of *mecA* designated as *mecA1* (Table 3). *mecA1* is considered to be the ancestry gene of *mecA* which historically did not have resistance towards oxacillin^35^. A recent study has shown that *S*. *sciuri* has developed oxacillin resistance using a variety of mechanisms from diversification of the non-binding domain of native PBPs, change in the *mecA* promoter, acquiring the SCC*mec* element and the adaptation of the bacterial genetic background^36^.

In this study we found that there was a large diversity of antibiotic resistant genes encoding resistance to different antibiotics. Of these genes, we found *blaZ* (90.7%) and *qacA/B* (51.16%) were the most common. Previous studies on the prevalence of antimicrobial resistance genes in CoNS from clinical and environmental sources are limited but some reports have shown that *blaZ* is of one the most common antibiotic resistance genes found in staphylococci^37,38^. *QacA/B* has been previously reported to high prevalence from University campus in Thailand (60.4%). This gene has an important role for the survival of the bacteria within the environment as they encode multidrug efflux pump which has shown cross resistance-towards antiseptic and disinfectant compounds used to reduce bacterial contamination from surfaces^39^.

In addition, we also found that *S*. *epidermidis* isolates were quite similar in their antibiotic resistance profile from our hierarchy clustering analysis even if they came from different areas. This may be due to that all isolates had the fluoroquinolone efflux pump gene *norA* and trimethoprim resistance dihydrofolate reductase gene *dfrC*^40,41^. It is possible that these genes are essential for *S*. *epidermidis* survival, especially as *norA* like *qacA/B* has shown reduce susceptibility to antiseptic and disinfectant substances.

SCC*mec* was detected in 36 out of the 49 isolates that were whole genome sequenced, however SCC*mec* types were assigned only to 17 isolates. The most common type was SCC*mec* type IV (n = 11), followed by SCC*mec* type V (n = 5) and SCC*mec* type VIII (n = 1). These results are consistent with previously reported studies of clinical and environmental isolates^11^. In our study SCC*mec* type IV was exclusively found in *S*. *epidermidis* isolates. This is in keeping with others reporting a high association between SCC*mec* type IV and *S*. *epidermidis*^42^. SCC*mec* type V was associated with *S*. *haemolyticus* and *S*. *warneri* isolates but is mainly reported to be associated with *S*. *haemolyticus* in clinical isolates^43^.

The remaining SCC*mec* types were untypeable as they harboured a novel *ccr* complex or multiple *ccr* complexes. Multiple *ccr* complexes have previously been described in clinical and community associated isolates^42^ but to the best of our knowledge, this is the first report of such SCC*mec* types determined in isolates recovered from the general public environments. It has been reported that multiple *ccr* complexes have shown to produce more stable *mecA* mRNA transcription compared to single elements as well as having a better cell wall integrity^42^. This suggests that isolates with multiple *ccr* complexes may have increased susceptibilities to oxacillin or cefoxitin, however not always correlate with their phenotypic data. It is possible that this adaptation helps the bacteria to survive longer periods under persistent antibiotic pressure.

MLST data showed a wide range of genetic variability among *S*. *epidermidis* isolates. ST2 was the most common sequence type identified which was consistent with previous reports studying multidrug resistant clinical isolates^24,25,44^. Although in our study isolates that harboured ST2 sequence types were isolated from public areas in hospitals, others reported that this sequence type was widely disseminated in clinical isolates recovered from patients^24,44,45^. In addition, in this study two new sequence types designated as ST771 and ST779 were identified in isolates recovered from a hospital in West London.

In conclusion, general public areas and common public areas in hospitals in London can be reservoirs for multidrug resistant staphylococci. These multidrug resistant bacteria can be found at high levels on high-frequency touched surfaces. A diverse range of SCC*mec* types were determined from general public settings and public areas in hospitals of which many were untypeable due to having either a novel *ccr* or an extra *ccr* complex. These SCC*mec* structures have not been previously reported in isolates recovered from environmental surfaces in general public settings.

Additional comparative genomics analyses are being conducted to decipher the genetic features of multidrug resistant staphylococci recovered from general public settings and to further our understanding of the origin and evaluation of these isolates.

## Materials and Methods

### Sample collection and screening of staphylococcal isolates

Samples were recovered from high-frequency hand touched surfaces of inanimate objects (door handles, stair handrails, toilet flushers, toilet seats, taps, lift buttons, chair armrests) from four locations in general public settings, two locations from East London and two locations from West London. Public settings included shopping centres (concourses, escalators lifts, public washrooms) and train stations (entry gates, public washrooms, escalators). Isolates were also recovered from a hospital setting where the general public had easy access, without being a patient or visiting a patient (reception area, public washrooms, corridors, lifts) (Table S1). From each location, 50 areas were randomly sampled using COPAN dry swabs (Copan Diagnostics Inc., USA). In total 600 isolates were screened of which 224 were from East London and 376 from West London. 182 of the isolates were from the community area and 418 from Hospital area. 97 from East London community area and 85 from West London community. 224 from East London hospital and 376 from West London hospital.

All samples were inoculated onto mannitol salt agar (MSA, Oxoid Basingstoke, UK) within 1–3 hours of recovery and incubated aerobically for 24–72 hours at 37 °C. The colonies were then screened for potential staphylococci characteristics, including performing catalase and coagulase tests. Prolex™ staph latex kits (ProLab Diagnostics, Neston, UK) was used to distinguish *S*. *aureus* and coagulase-negative *Staphylococcus*.

### Antimicrobial Susceptibility testing

The samples were tested for their susceptibility against a panel of 11 antibiotics by using a standard disc diffusion method^46^. The antibiotics tested were the following: oxacillin (1 µg), gentamicin (10 µg), mupirocin (20 µg), amoxicillin (10 µg), erythromycin (15 µg), tetracycline (10 µg), cefoxitin (30 µg), cefepime (30 µg), fusidic acid (10 µg), penicillin (1 unit) and chloramphenicol (30 µg) (Mast Group, Merseyside, UK). Antibiotic profiles of each isolate ware determined according the recommendation of the Clinical & Laboratory Standards Institute (CLSI) and British Society for Antimicrobial Chemotherapy (BSAC)^46,47^.

In addition, the minimum inhibitory concentrations (MIC) for oxacillin and cefoxitin were determined using E-tests (Biomerieux, Basingstoke, UK)^46,47^.

### Identification of multidrug-resistance staphylococci recovered from high-frequency hand touch areas

Potential staphylococci isolates were initially identified by conventional methods, including gram staining catalase and coagulase tests. All the isolates were identified at species level using Matrix-assisted laser desorption ionization time of flight mass-spectroscopy (MALDI-TOF-MS, Microflex LT, Bruker Daltonics, Coventry, UK) in a positive linear mode (2000–20,000 m/z range). Samples were prepared as described previously^7^. MALDI-TOF Biotyper 3.0 software (Bruker Daltonics, Coventry, UK) was used to analyse the spectra and to identify the bacterial species. Bacterial test standard *Escherichia coli* DH5α (Bruker Daltonics, Coventry, UK) was used for calibration and as a standard for quality control.

### Detection of *mecA* gene

The detection of the *mecA* gene was carried out by PCR for all staphylococci isolates. Freshly grown samples were suspended into 40 µl of sterile distilled water and boiled at 100 °C then cooled on ice for 5 minutes. The samples were then centrifuged at 13,000 × g for 1 minute and the supernatant was used for the PCR providing the DNA template.

The PCR was performed using Met1 and Met2 primers as described previously^48^. PCR reactions were performed in a 20 µl volume for each sample which consists of 10 µl of Phusion Master Mix;1 µl of met1, 1 µl of met2, 6 µl of sterile distilled water and 1 µl of isolates DNA template. The PCR condition for this reaction was 94 °C for 5 minutes followed by 35 cycles of denaturation at 94 °C for 30 seconds, annealing at 52 °C for 30 seconds and extension at 72 °C for 1 minute with a final extension at 72 °C for 10 minutes.

### WGS and bioinformatic analyses

Forty-nine staphylococci *mecA* positive isolates were whole genome sequenced using Illumina HiSeq platform. Thirteen out of 49 isolates were whole genome sequenced by MicrobesNG (Birmingham, UK) and the remining isolates were sequenced at Fudan University, Shanghai, China.

Genomic DNA was extracted using TIANamp Bacteria DNA kit (Tiangen, China) and paired-end sequencing libraries were constructed using Nextera XT DNA Sample Preparation kits or TruSeq DNA HT Sample Prep Kit (Illumina, USA) following manufacturer’s instruction. The short read sequencing data were deposited in the European Nucleotide Archive, under the study PRJEB30498. The accession numbers for individual samples are included in Supplementary data (Table S2).

The raw reads quality was assessed using FASTQC and trimmed using trimmomatic (Version 0.35), default settings, specifying a phred cutoff of Q20^49,50^. The trimmed reads were de novo assembly by SPAdes 3.11 and annotated by Prokka 1.12^51,52^.

The species of these isolates were confirmed by 16sRNA sequencing^53^. 16S rRNA sequences were extracted from assembled genomes using the barrnap software (https://github.com/tseemann/barrnap) and searched against a database of known 16S ribosomal RNA sequences using NCBI BLAST tool with a cutoff for species identity of 95% similarity^54^.Antibiotic resistance genes were detected using the Comprehensive Antibiotic Resistance Database (CARD) server^55^.

The diversity of SCC*mec* types were determined by searching against a database of known *SCCmec* molecular markers with NCBI BLAST with a cutoff e-value of 1e-5^54,56^.

*S*. *epidermidis* isolates were analysed by Multi locus sequence typing (MLST) and the sequence types for each isolate were assigned using MLST2.0 online service (https://cge.cbs.dtu.dk/services/MLST/)^57^.

### Statistical analysis

A Chi-squared test was performed to identify any significant difference in the proportion of multidrug resistant staphylococci and *mecA* gene in isolates recovered from general public settings and public areas in hospitals in East and West London^58^. A P value of >0.05 was considered to be significant. Barnard Exact test was performed to identify significance in the proportion of antibiotic resitance genes form WGS sample recovered from general public settings and public areas in hospitals in East and West London^59^. A two side P value of >0.05 was considered to be significant. Hirachy clustering of a heatmap for resistance gene phentype and genotype were created using the R package ‘Heatmap.plus’ (https://cran.r-project.org/web/packages/heatmap.plus/index.html).

## Supplementary information

Supplementary Information

## Data Availability

All data generated or analysed during this study are included in this published article and its supplementary information files.
