# Supplementary material for: Whole genome sequencing revealed new molecular characteristics in multidrug resistant staphylococci recovered from high frequency touched surfaces in London
Source: Sci Rep. 2019 Aug 1;9:9637. doi: 10.1038/s41598-019-45886-6 (PMC6675788; doi:10.1038/s41598-019-45886-6)
Supplement: Supplementary file 1 — Supplementary Information [file 41598_2019_45886_MOESM1_ESM.pdf]

Whole genome sequencing revealed new molecular characteristics in multidrug resistant staphylococci recovered from high frequency touched surfaces in London

Rory Cave[1]; Raju Misra[2]; Jiazhen Chen[3]; Shiyong Wang[3]; Hermine V Mkrtchyan[1*]

[1]University of East London, School of Health, Sport and Bioscience, Water Lane, London, E15 4LZ

[2]Natural History Museum, Core Research Laboratories, Molecular Biology, Cromwell Rd, London SW7 5BD

[3]Department of Infectious Disease, Huashan Hospital, Fudan University, 12 Middle Wulumuqi Rd., Shanghai 200040, China

*H.Mkrtchyan@uel.ac.uk

Supplementary Information

Table S1: Summary of the environmental sites in hospital and community general public areas

	General Public Areas			
	ELC	WLC	ELH	WLH
Specific sites	Wash room door handles<br>Washroom taps<br>Toilet flusher<br>Toilet seat<br>Soap dispensers<br>Door handles<br>Elevator button<br>Bench arm rest<br>Escalator rail<br>Stair rail<br>ATM machines<br>Ticket machine<br>Pedestrian crossing buttons	Wash room door handles<br>Washroom taps<br>Toilet flusher<br>Toilet Seat<br>Soap dispensers<br>Door handles<br>Elevator Button<br>Bench Arm rest<br>Escalator rail<br>Stair rail<br>ATM machines<br>Ticket machine<br>Touch screen TV<br>Public phone<br>Pedestrian crossing buttons	Wash room door handles<br>Washroom taps<br>Toilet flusher<br>Toilet seat<br>Soap dispensers<br>Door handles<br>Elevator button<br>Seat arm rest<br>Stair hand rail	Wash room door handles<br>Washroom taps<br>Toilet flusher<br>Toilet seat<br>Soap dispensers<br>Door handles<br>Elevator Button<br>Seat arm rest<br>Stair hand rail<br>Baby changing area
Number of isolates	97	85	127	291

ELC= East London Community; WLC=West London Community; ELH=East London Hospital, WLH=West London Hospi

Table S2. Summary of the Whole Genome Sequenced isolates			
Acssion No.	Sample No	Species	Area isolated
ERS2999996	1	Staphylococcus haemolyticus	East London Community
ERS2999997	27	Staphylococcus sciuri	East London Community
ERS2999998	33	Staphylococcus sciuri	East London Community
ERS2999999	59	Staphylococcus sciuri	East London Community
ERS3000000	74	Staphylococcus sciuri	East London Community
ERS3000001	75	Staphylococcus sciuri	East London Community
ERS3000002	93	Staphylococcus haemolyticus	East London Community
ERS3000003	99	Staphylococcus haemolyticus	East London Community
ERS3000004	105	Staphylococcus haemolyticus	East London Community
ERS3000005	109	Staphylococcus sciuri	East London Community
ERS3000006	207	Staphylococcus hominis	West London Community
ERS3000007	208	Staphylococcus hominis	West London Community
ERS3000008	209	Staphylococcus hominis	West London Community
ERS3000009	211	Staphylococcus cohnii	West London Community
ERS3000010	321	Staphylococcus epidermidis	East London Hospital
ERS3000011	327	Staphylococcus epidermidis	East London Hospital
ERS3000012	329	Staphylococcus epidermidis	East London Hospital
ERS3000013	343	Staphylococcus cohnii	East London Hospital
ERS3000014	349	Staphylococcus cohnii	East London Hospital
ERS3000015	355	Staphylococcus epidermidis	East London Hospital
ERS3000016	361	Staphylococcus haemolyticus	East London Hospital
ERS3000017	372	Staphylococcus hominis	East London Hospital
ERS3000018	373	Staphylococcus haemolyticus	East London Hospital
ERS3000019	385	Staphylococcus hominis	East London Hospital
ERS3000020	386	Staphylococcus hominis	East London Hospital
ERS3000021	387	Staphylococcus hominis	East London Hospital
ERS3000022	407	Staphylococcus epidermidis	East London Hospital
ERS3000023	435	Staphylococcus epidermidis	West London Hospital
ERS3000024	436	Staphylococcus haemolyticus	West London Hospital
ERS3000025	445	Staphylococcus haemolyticus	West London Hospital
ERS3000026	465	Staphylococcus epidermidis	West London Hospital
ERS3000027	475	Staphylococcus epidermidis	West London Hospital
ERS3000028	479	Staphylococcus hominis	West London Hospital
ERS3000029	492	Staphylococcus haemolyticus	West London Hospital
ERS3000030	506	Staphylococcus haemolyticus	West London Hospital
ERS3000031	538	Staphylococcus haemolyticus	West London Hospital
ERS3000032	620	Staphylococcus hominis	West London Hospital
ERS3000033	623	Staphylococcus hominis	West London Hospital
ERS3000034	631	Staphylococcus epidermidis	West London Hospital
ERS3000035	664	Staphylococcus epidermidis	West London Hospital
ERS3000036	673	Staphylococcus epidermidis	West London Hospital
ERS3000037	699	Staphylococcus warneri	West London Hospital
ERS3000038	700	Staphylococcus warneri	West London Hospital
ERS3000039	702	Staphylococcus warneri	West London Hospital
ERS3000040	711	Staphylococcus epidermidis	West London Hospital
ERS3000041	712	Staphylococcus epidermidis	West London Hospital
ERS3000042	713	Staphylococcus epidermidis	West London Hospital
ERS3000043	715	Staphylococcus epidermidis	West London Hospital
ERS3000044	716	Staphylococcus epidermidis	West London Hospital
